# Molecular Detection of *Theileria equi*, *Babesia caballi*, and *Borrelia burgdorferi* Sensu Lato in *Hippobosca equina* from Horses in Spain

**DOI:** 10.3390/pathogens15010094

**Published:** 2026-01-15

**Authors:** Abel Dorrego, Sergi Olvera-Maneu, Eduard Jose-Cunilleras, Paloma Gago, Alejandra Raez, Belen Rivera, Ariana Oporto, Sergio Gonzalez, Fatima Cruz-Lopez

**Affiliations:** 1 VISAVET Health Surveillance Centre, Universidad Complutense, 28040 Madrid, Spain; abeldorr@ucm.es (A.D.); goser@ucm.es (S.G.); 2 Department of Veterinary Medicine, School of Veterinary Medicine, University of Nicosia, 2414 Nicosia, Cyprus; olvera-maneu.s@unic.ac.cy; 3 Department of Animal Medicine and Surgery, Universitat Autònoma de Barcelona, 08193 Barcelona, Spain; 4 Animal Health Department, Facultad de Veterinaria, Universidad Complutense, 28040 Madrid, Spain

**Keywords:** *Borrelia*, Diptera, equine piroplasmosis, haemoparasites, *Hippobosca equina*, PCR, phylogenetics, vector-borne pathogens

## Abstract

The forest fly *(Hippobosca equina*) is an obligate haematophagous dipteran insect (order Diptera) that primarily infests horses and may contribute to the circulation of vector-borne pathogens. This study aimed to investigate the presence of *Anaplasma phagocytophilum*, *Borrelia burgdorferi* s.l., *Babesia caballi*, and *Theileria equi*, important vector-borne pathogens of equids, in forest flies collected from horses in endemic areas of Spain. A total of 170 forest flies were collected from 39 equids across four geographical regions in Spain (Segovia, Madrid, Toledo, and Menorca) and blood samples were collected from 27 of these horses. All flies were morphologically and molecularly identified as *H. equina*, and DNA extracted from flies and equine blood was screened using multiplex real-time and nested PCR, followed by sequencing and phylogenetic analysis. Neither flies nor horses tested positive for *A. phagocytophilum*, whereas one fly was positive for *B. burgdorferi* s.l. (0.6%). In contrast, *T. equi* and *B. caballi* DNA were detected in 11.2% and 1.2% of flies, respectively, and all positive flies were collected from horses positive for equine piroplasmosis (*T. equi*/*B. caballi* infection), with identical 18S rRNA sequences between hosts and flies. Nested PCR showed a higher detection rate than real-time PCR for the detection of these piroplasms in flies and blood samples. These findings provide the first molecular evidence of EP pathogens in *H. equina* and support further investigation into the epidemiological importance of forest flies in equine pathogen surveillance.

## 1. Introduction

Hippoboscid flies, commonly known as louse flies, are obligate haematophagous dipterans (order Diptera) that infest mammals and birds worldwide [1]. Several species of louse flies within the genus *Hippobosca*, particularly those infesting horses (*Hippobosca equina*) and dogs (*Hippobosca longipennis*), bite their hosts and caretakers, causing discomfort, pruritus, and, in some cases, anaphylactic shock requiring emergency treatment in humans [2,3,4].

*H. equina*, commonly known as the forest fly, primarily affects horses, but has also been reported on cattle, dogs, hares, birds, and even humans [4,5]. It feeds on blood and is active on warm days, targeting sensitive areas such as the perineum, udder, and inner thighs of horses [5]. *H. equina* is a periodic parasite, feeding briefly on a host before departing to either find another host or move to the ground to deposit third-instar larvae (pre-pupae) [6]. This species has evolved specialized morphological and physiological adaptations for its parasitic lifestyle, including leg claws for gripping and piercing-sucking mouthparts that enable it to feed on blood multiple times daily, often ranging from several to over 10 feedings per day [6]. Unlike other hippoboscids, its wings remain functional throughout its lifespan [7], a trait that facilitates its preference for parasitizing short-haired animals and frequent host-switching [8]. *H. equina* is most frequently reported in warm climatic regions, where it represents an ecologically relevant ectoparasite [9]. Through its effects on host welfare and behaviour, this species may alter animal activity, grazing behaviour, and reproductive performance, with potential downstream consequences for host population structure and broader ecological relationships [9]. Furthermore, the forest fly is considered both a mechanical and biological vector of various pathogens, including *Corynebacterium pseudotuberculosis* [10]. *Hippobosca equina* has been implicated in the transmission of a wide range of bacterial, viral, and protozoan agents affecting horses as well as domestic and wild ruminants. These include species of *Anaplasma* [11,12], *Bartonella* [13,14,15], *Borrelia* [11], bluetongue virus 9 [16], bovine viral diarrhoea virus type 2 [16], and *Trypanosoma* spp. [14].

Both *Anaplasma phagocytophilum* and *Borrelia burgdorferi* s.l. are vector-borne bacterial pathogens of increasing concern in equine health in Europe, including Spain [17,18,19,20,21]. *A. phagocytophilum*, the causative agent of equine granulocytic anaplasmosis, is an obligate intracellular bacterium that infects neutrophils and is primarily transmitted by *Ixodes* ticks [17]. Clinical signs in horses range from fever and lethargy to ataxia and limb oedema [17]. *B. burgdorferi* s.l., a spirochete responsible for Lyme borreliosis, is also transmitted by *Ixodes* ticks and has been associated with lameness, neurological disorders, and poor performance in horses [22]. Both pathogens are relevant not only because of their impact on equine health but also due to their zoonotic potential [17,22].

Equine piroplasmosis (EP) is a tick-borne protozoan disease affecting equids [23], caused by three intra-erythrocytic haemoprotozoans: *Babesia caballi*, *Theileria equi* and the recently identified species *Theileria haneyi* [24]. In this study, the term EP is used to refer to infection with these piroplasms, irrespective of the presence of clinical signs, as most infected horses remain asymptomatic carriers. Transmission occurs predominantly via ixodid ticks, with species belonging to the genera *Dermacentor*, *Hyalomma*, and *Rhipicephalus* playing a major role in pathogen spread [25,26]. This disease has an important impact on animal welfare, as well as the global equine industry and trade [27,28]. Although *Theileria ovis* and *Theileria luwenshuni* have been detected in *Melophagus ovinus* and *Lipoptena fortisetosa*, respectively [29,30,31], there are no studies to date reporting the detection of any piroplasms in *H. equina*.

Considering the impact of *A. phagocytophilum* and *B. burgdorferi* s.l. on horse health and their potential to cause zoonotic diseases, as well as the significant effect of equine piroplasmosis on the horse industry, there is a need for ongoing surveillance of vector populations in Spain. This study aimed to detect these vector-borne blood pathogens in forest flies infesting horses in endemic regions of Spain using molecular techniques.

## 2. Materials and Methods

### 2.1. Study Area and Collection of Samples

**Figure 1:**
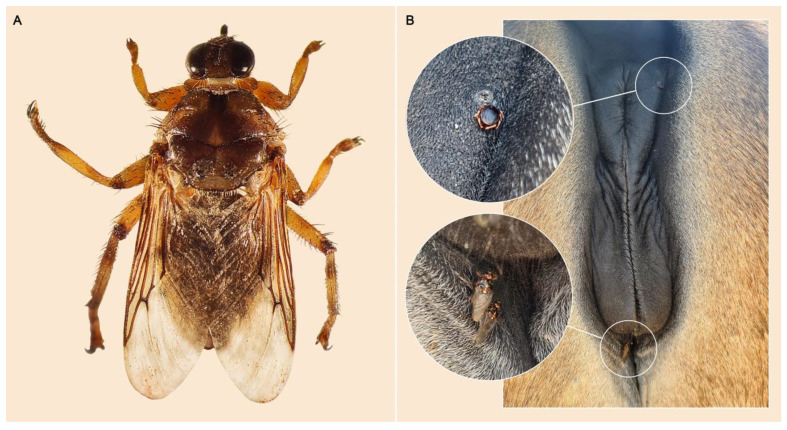
Representative specimen of *Hippobosca equina* collected during the present study (**A**), and perineal region of a mare showing a mixed infestation with *Hyalomma marginatum* and *H. equina* (**B**).

Sampling was conducted on a convenience basis in endemic areas selected for the presence of *H. equina* and a high incidence of equine vector-borne diseases. Hippoboscid flies were collected from horses (*Equus caballus*; n = 39) and one mule (*Equus caballus* × *Equus asinus*) across four provinces in Spain (Segovia, Madrid, Toledo, and Menorca) between September 2021 and August 2024. A total of 170 forest flies were manually collected directly from the perineum and inner thighs of the examined animals. The flies were carefully removed from the skin and immediately stored in 70% ethanol in individual Eppendorf tubes. All hippoboscids were identified to the species level using a stereomicroscope and morphological keys [32]. Figure 1 shows a representative *H. equina* individual collected in this study (A) and the perineum of a mare with a mixed infestation of *Hyalomma marginatum* and *H. equina* (B).

In the provinces of Segovia and Menorca, blood samples were obtained from a subset of the examined equids (27 horses) by the attending veterinary surgeon, who collected 5 mL of blood from the jugular vein. Blood samples were collected by veterinarians as part of routine clinical screening, with informed owner consent, and surplus material was used for research purposes. Collected blood was distributed into two tubes, one containing EDTA and a second tube without anticoagulant. Four horses (horses 6, 9, 10, and 14) were sampled on two separate occasions. All blood samples were obtained during routine veterinary examinations and were used for EP screening.

Blood samples were maintained under refrigerated conditions and promptly transported to the laboratory. Upon arrival, tubes without anticoagulant were centrifuged at 600× *g* for 5 min to obtain serum. Serum samples and EDTA-treated whole blood were then stored at −80 °C until serological analysis and DNA extraction, respectively.

### 2.2. Genomic DNA Extraction

Genomic DNA was isolated from individual hippoboscid flies for molecular detection of *A. phagocytophilum*, *B. burgdorferi* s.l, *B. caballi*, and *T. equi*, as well as for insect species identification by DNA barcoding. DNA extraction was performed using the QIAamp DNA Mini Kit (QIAGEN, Madrid, Spain) following the manufacturer’s recommended protocol.

EDTA blood samples from horses were thawed, vortexed to ensure homogenization, and subsequently used for molecular screening of the same pathogens, with total genomic DNA extracted using the same kit according to the manufacturer’s protocol.

### 2.3. PCR Assay for Insect Identification

To confirm the species identity and explore the genetic diversity of the collected hippoboscids, total DNA extracted from 28 forest flies was screened to amplify a 648 bp fragment of the mitochondrial COI (Cytochrome c oxidase subunit I) gene by conventional PCR using the primers listed by Hebert et al. [33]. The PCR products were sequenced by Sanger at Macrogen Spain (Madrid, Spain), and the resulting COI sequences were used for taxonomic identification by comparison with available hippoboscid sequences in the GenBank nucleotide database using BLASTn (Basic Local Alignment Search Tool) (https://blast.ncbi.nlm.nih.gov/Blast.cgi) (accessed on 14 November 2024) with default parameters and BOLD (Barcode of Life Data Systems) (www.boldsystems.org) (accessed on 14 November 2024).

### 2.4. PCR Assay for A. phagocytophilum and B. burgdorferi Screening

DNA samples obtained from equine blood and individual hippoboscid flies were analysed using a multiplex real-time PCR approach targeting *A. phagocytophilum* and *B. burgdorferi* s.l. The assay design, including primer and probe sequences as well as amplification conditions, was based on the protocol previously described by Courtney et al. [34]. Sequence-confirmed positive clinical samples were used as positive controls for the detection of *A. phagocytophilum* and *B. burgdorferi* sensu lato, and molecular-grade water was used as a negative control in each PCR run. Any positive samples to *B. burgdorferi* by real-time PCR were later tested using a conventional PCR with primers LDF and LDR as described by Marconi and Garon [35].

### 2.5. PCR Assay for Equine Piroplasms Screening

The presence of equine piroplasms was investigated in DNA samples obtained from horse blood and individual hippoboscid flies by means of a duplex real-time PCR approach. The assay targeted *T. equi* and *B. caballi*, and was implemented using previously validated oligonucleotides and cycling parameters described by Camino et al. [36]. Two equine blood samples previously characterized as positive for *T. equi* or *B. caballi* by PCR and confirmed by Sanger sequencing of the 18S rRNA gene in a previous study [37] were used as positive controls. RNase-free water was included as a negative control in each PCR run.

### 2.6. Nested PCR Assay for Equine Piroplasms Characterization

All DNA extractions (170 hippoboscid flies and blood samples from 27 horses) were analysed using a nested PCR targeting an ~800 bp fragment of the 18S rRNA locus for the genera *Babesia* and *Theileria*, with primers and conditions as described by Jefferies et al. [38]. Amplicons were visualized on an agarose gel, and products of the expected size were purified using the QIAquick PCR Purification Kit (QIAGEN, Madrid, Spain) before being sent to Macrogen Spain (Madrid, Spain) for Sanger sequencing. Species identification of the sequences obtained was performed using the Basic Local Alignment Search Tool (BLAST) and comparison with sequences deposited in the non-redundant National Center for Biotechnology Information (NCBI) database (https://blast.ncbi.nlm.nih.gov/) (accessed on 24 October 2024). The MUSCLE (Multiple Sequence Comparison by Log-Expectation) function [39], within the AliView alignment viewer and editor [40] was used to align and compare the study sequences with those previously determined and deposited in GenBank for *T. equi* and *B. caballi*. To assess the genetic diversity of both hemoparasites within the study samples, species-specific Neighbour-Joining phylogenetic trees were constructed using MEGA 7.0.26 software [41]. The 18S SSU rRNA gene sequences of *Theileria parva* (L02366) and/or *Theileria annulata* (KX375830) were included in the trees as outgroups. Representative sequences for the distinct *T. equi* and *B. caballi* clades detected in this study were submitted to the NCBI GenBank database (accession numbers PQ498866 and PQ498874 for *T. equi* isolates, and PQ498881 and PQ508386 for *B. caballi*).

### 2.7. Equine Piroplasmosis Serology

Serum samples were thawed and tested for equine IgG-specific antibodies against *T. equi* and *B. caballi* using two commercial competitive ELISA (cELISA) kits [*Theileria equi* and *Babesia caballi* Antibody Test Kits (VMRD^®^ Inc., Pullman, WA, USA)] according to the manufacturer’s instructions. Results were calculated from the optical density measurements at 620 nm and expressed as inhibition percentages (IP). Sera were classified as positive (IP ≥ 40%) or negative (IP < 40%).

### 2.8. Data Analysis

Data processing and descriptive statistics were performed using Microsoft Excel and IBM SPSS Statistics version 25 (IBM Corporation, Armonk, NY, USA). Proportions are presented with exact 95% confidence intervals calculated using the Clopper–Pearson method. Differences in paired detection frequencies of *T. equi* and *B. caballi* in flies were assessed using McNemar’s exact test (two-sided).

## 3. Results

### 3.1. Identification and DNA Barcoding of Collected Hippoboscid Flies

All collected hippoboscids were identified as *H. equina* using a stereomicroscope, taxonomic keys, and mitochondrial COI sequencing. Barcode analysis of 58 individual *H. equina* specimens in BOLD revealed that their COI sequences clustered within a respective Barcode Index Number, matching sequences previously reported from Europe (Portugal, Corsica, Slovenia, Austria) (BOLD: AAX0882). All 58 sequences were identical, and a representative COI sequence obtained in this study was submitted to GenBank under accession number PQ608715.

### 3.2. A. phagocytophilum and B. burgdorferi Screening—Hippoboscid Flies and Horses

All hippoboscid flies and horses tested negative for *A. phagocytophilum* by real-time PCR. Regarding *B. burgdorferi*, molecular screening revealed a positive in one of the collected forest flies (a specimen collected in Horse 3, in Segovia, in September 2021). The *Borrelia*-positive specimen yielded a high Cq (Quantification Cycle) value (Cq = 37) in the real-time PCR assay but tested negative when re-analysed using conventional PCR.

### 3.3. Equine Piroplasms Screening—Hippoboscid Flies

In forest flies (n = 170), real-time PCR detected *T. equi* DNA in 11 samples (6.5%; 95% CI: 3.3–11.3), whereas nested PCR detected *T. equi* DNA in 19 samples (11.2%; 95% CI: 6.9–16.9). For *B. caballi,* real-time PCR did not detect any positive samples (0/170; 0.0%; 95% CI: 0.0–2.2), while nested PCR identified two positive samples (2/170; 1.2%; 95% CI: 0.1–4.2).

The *T. equi* DNA–positive flies were collected from three horses in the province of Segovia (horses 1, 6 and 14) and one horse in the province of Toledo (horse 4). The positive *B. caballi* DNA flies were collected from two horses in Segovia (horses 7 and 14). The horses with positive EP flies, their area of residence, sampling dates, total number of flies collected, and the number of positive flies per horse are shown in Table 1. A spatial overview of the sampling sites and nested PCR results for equine piroplasmosis (EP) in forest flies and horses is provided in Supplementary Figure S1.

### 3.4. Equine Piroplasms Screening—Horses

Of the horses examined in Segovia (n = 7), five were positive for piroplasm infection by both real-time and nested PCR. These included two horses infected with *T. equi*, two with *B. caballi*, and one animal (Horse 14) that showed a change in infection status, testing positive for *B. caballi* at the initial sampling and for *T. equi* three months later. In Menorca (n = 20), *T. equi* DNA was identified in eight horses by nested PCR (40.0%; 95% CI: 19.1–63.9), whereas real-time PCR detected *T. equi* DNA in four horses (20.0%; 95% CI: 5.7–43.7).

Regarding EP serology, all horses sampled in Segovia (n = 7) tested positive for either *T. equi*, *B. caballi*, or both at some point during the study. Only one horse (horse 10) became seronegative for *B. caballi* after eleven months. Horse 14 (positive for *B. caballi* at one time point and positive for *T. equi* three months later) showed positive results for both *T. equi* and *B. caballi* at both time points. Of the horses sampled on Menorca (n = 20), 12 were positive for T. equi by cELISA.

The results for EP real-time PCR, nested PCR, EP clade, and cELISA, along with the characteristics of each sampled horse (sex, residence area, and sampling date), are presented in Supplementary Table S1. Sampled horses with positive EP flies are marked with an asterisk after their identification. Horse 6 tested positive for *T. equi* by real-time PCR, nested PCR, and cELISA in 2023 and 2024. Positive *T. equi* DNA was found in flies collected from this horse at three time points during these two years. Horse 7 was positive for *B. caballi* by real-time PCR, nested PCR and cELISA, and at least one fly collected at the same time point tested positive for *B. caballi*. Finally, horse 14 tested positive for *B. caballi* in May 2024, when a positive *B. caballi* fly was also collected from it. However, it tested positive for *T. equi* in August 2024, with positive *T. equi* flies collected at three different time points in July and August 2024.

### 3.5. Equine Piroplasmosis Characterization and Phylogenetic Analysis

**Figure 2:**
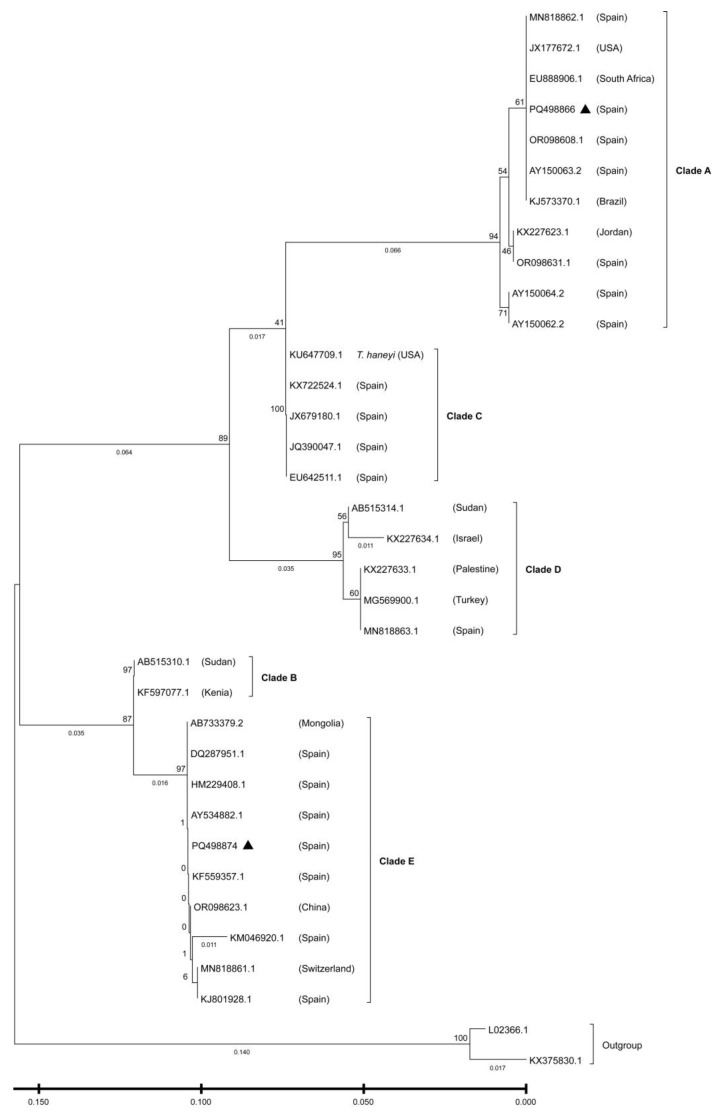
Maximum likelihood tree of *Theileria equi* isolates based on sequences from the amplified 18S SSU rRNA V4 hypervariable region. *T. equi* sequences reported previously are denoted by their GenBank accession numbers and countries of isolation. Bold triangles indicate sequences obtained in the present study. Numbers above the branches represent bootstrap values (1000 replications). Previously described clades (**A**–**E**) are highlighted.

**Figure 3:**
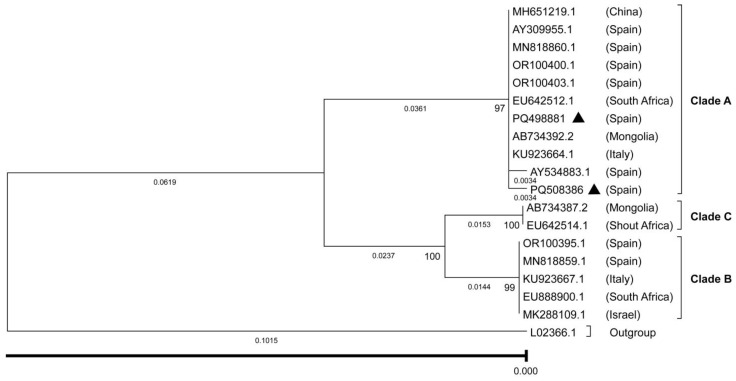
Maximum likelihood tree of *Babesia caballi* isolates based on sequences derived from the amplified 18S SSU rRNA V4 hypervariable region. *B. caballi* sequences reported previously are denoted by their GenBank accession numbers and countries of isolation. Bold triangles represent sequences obtained in the present study. Numbers above the branches correspond to bootstrap values (1000 replications). Previously described clades (**A**–**C**) are highlighted.

EP-positive flies and their equine hosts resulted in identical sequences in all cases. Within the sequences obtained in this study, two distinct *T. equi* (GenBank accession numbers PQ498866, PQ498874) and two *B. caballi* sequences (PQ508386, PQ498881) were identified. Sequence identification was based on BLASTn comparisons, with all sequences showing query coverage of ≥99%, nucleotide identity of ≥98% with reference sequences, and E-values of 0.0. Phylogenetic analysis of *T. equi* (Figure 2) showed that the sequences obtained in this study clustered within two major groups. One sequence grouped within clade A (PQ498866), together with reference isolates from Spain, South Africa, the USA, Brazil, and Jordan, while another clustered within clade E (PQ498874), alongside isolates from Spain, Switzerland, Saudi Arabia, Mongolia, Korea, and China. In both cases, sequence identity with reference isolates from the corresponding clades was high (approximately 99%). Similarly, the phylogenetic tree inferred for *B. caballi* (Figure 3) placed the sequences within clade A, clustering with isolates from Spain, South Africa, Mongolia, Italy, and China, and showing high nucleotide identity with sequences from this clade (99.9% for PQ508386 and 100% for PQ498881).

## 4. Discussion

In the present study, no evidence of *A. phagocytophilum* DNA was detected in either *H. equina* specimens or in the examined horses. This finding contrasts with previous reports detecting *Anaplasma* DNA in *M. ovinus* and *H. equina* [11,12]. Although a seroprevalence of 6.5% for *A. phagocytophilum* has been reported in horses from Galicia, in north-western Spain [21], the central region sampled in the present study may exhibit a much lower prevalence. Similar north–south gradients have been described for other tick-borne diseases, such as EP, and a reduced local prevalence could therefore explain the absence of *A. phagocytophilum* detection in our samples.

Conversely, molecular analysis revealed one *H. equina* specimen positive for *B. burgdorferi* s.l., supporting earlier evidence of the molecular detection of this bacterial pathogen in forest flies [11]. Unfortunately, the *Borrelia* real-time PCR–positive sample showed a high Cq value (Cq = 37) and was negative by conventional PCR, suggesting a low DNA concentration near the analytical detection limit, therefore it could not be sequenced. No studies to date have demonstrated the vector competence of *H. equina* for *B. burgdorferi* s.l., and further investigations are therefore warranted to elucidate the epidemiological significance of this species in the transmission of bacterial pathogens to horses.

In addition to bacterial pathogens, our study also investigated the presence of protozoan agents responsible for EP. In contrast to the absence of *A. phagocytophilum* and the isolated detection of *B. burgdorferi* s.l., both *T. equi* and *B. caballi* were identified in dipteran specimens. While earlier studies reported the detection of *T. ovis* in *M. ovinus* and *T. luwenshuni* in *L. fortisetosa* [29,30], previous efforts to detect piroplasm DNA in *H. equina* had not been successful prior to the present work [11,14]. It should be noted that EP positivity in this study reflects molecular or serological evidence of infection rather than clinical disease, as none of the sampled horses showed signs compatible with acute equine piroplasmosis at the time of sampling.

In *H. equina* specimens, the proportion of samples positive for *T. equi* was greater than that observed for *B. caballi* (11.2% and 1.2%, respectively; McNemar’s exact test, *p* < 0.001). This finding aligns with the higher prevalence of *T. equi* compared to *B. caballi* in Spain (29.0% vs. 1.8%) [37]. The two regions in Spain where horses were sampled and tested for EP, Segovia and Menorca, are areas of high EP prevalence. Segovia, part of Castile-Leon community, showed a prevalence of 26.7% for *T. equi* and 5.5% for *B. caballi* [37]. Menorca, part of the Balearic Islands community, had a 0% prevalence in a recent nationwide study [37]. However, the horses sampled in this study were Pure Breed Menorcan Horses, and data from pre-exportation analyses conducted between 2021 and 2024 at the VISAVET Equine Health Surveillance Centre indicate a high EP serological exposure (54.6%) in these horses on Menorca (unpublished data). Sampling from these two regions likely increased the chances of detecting EP-positive *H. equina* specimens, in contrast to other studies that did not find any piroplasms in these flies [11,14].

Across both forest fly specimens and horse blood samples, nested PCR identified a greater number of positive cases than real-time PCR, detecting additional positive samples. This is consistent with studies reporting a higher sensitivity of the nested PCR [<10 parasitized erythrocytes (PE)] versus the real-time (10–100 PE) [42,43,44], and in fact the multiplex real-time PCR used in this study had a detection limit of 3.0 × 10^−4^% PE for *T. equi* and 2.0 × 10^−3^% PE for *B. caballi* [36] while the nested PCR had a detection limit of 2.7 × 10^−6^% PE for the primary round of amplification and 2.7 × 10^−7^% in the secondary round of amplification [38].

EP positivity in the flies collected for this study ranged from 5% to 100% (mean: 23.7%, median: 33.3%). This suggests that approximately one-quarter of the flies collected from an EP-positive horse would test positive for EP, making it relatively straightforward to detect an EP-positive horse by sampling the flies. While collecting forest flies from the perineal area of feral horses may pose some risks, this method is likely more practical than drawing blood samples in such populations, particularly when the goal is to detect EP.

All forest flies testing positive for EP were collected from horses that were also EP-positive at the time of sampling. In addition, in one case (Horse 14), the EP status detected in flies reflected the change in host infection from *B. caballi* to *T. equi* between sampling time points. Furthermore, the 18S SSU rRNA V4 region sequences of *T. equi*/*B. caballi* obtained from all the collected flies were identical to those recovered from their equine hosts. These findings suggest that the EP-positive flies likely fed on their hosts shortly before collection. However, since *H. equina* can feed on multiple hosts, it is possible that in horses sharing the same environment (e.g., Horses 4, 6, and 7), EP could originate from different horses [45].

The *T. equi* and *B. caballi* sequences recovered from both horses and flies in this study were distributed among the most prevalent clades reported in Spain. *T. equi* isolates from flies and horses in Segovia clustered in clade E, whereas *T. equi* isolates from flies and/or horses in Toledo clustered in clade A. *T. equi*–positive horses from Menorca clustered in clade A, except for one animal (horse 17), which clustered in clade E. These two clades are the most common in Spain, with clade E being widespread across the country and clade A predominantly found in southern and central Spain [46]. As for *B. caballi*, all isolates from horses and flies in Segovia in this study belonged to clade A, which aligns with the distribution of this clade in the northern regions of Spain [46].

Although ixodid ticks remain the only confirmed biological vectors of EP, the detection of piroplasm DNA in dipteran flies is consistent with earlier studies demonstrating their ability to transfer infected blood between hosts during interrupted feeding [47,48]. The haematophagous behaviour of these flies, coupled with their tendency to share hosts—and often feeding sites—with ticks (Figure 1B), underscores the need for experimental transmission studies to clarify their epidemiological role in piroplasm spread. Until vertical or mechanical transmission of EP by *H. equina* is experimentally confirmed, its involvement should be considered a possibility rather than an established route. Consequently, control strategies for horses in endemic areas should continue to focus on tick management through acaricides and routine grooming [26,37], but should also incorporate measures targeting flies, including the use of insecticides or repellents and regular inspection of the perineum, udder, and inner thighs to detect and manage forest fly infestations.

Although vector competence cannot be established from DNA detection alone, the identification of EP pathogens—and the detection of *B. burgdorferi* s.l. in one specimen—together with previous reports of *H. equina* acting as a mechanical and biological vector of other pathogens such as *Corynebacterium pseudotuberculosis* [10], provide a strong rationale for further investigation into the broader vectorial capacity of this species in equine environments.

## 5. Conclusions

This study provides the first molecular evidence of *T. equi* and *B. caballi* in *H. equina* collected from horses, with a strong correspondence between the piroplasm status of flies and their hosts. While *A. phagocytophilum* was not detected and only one specimen tested positive for *Borrelia burgdorferi* sensu lato, the identification of equine piroplasms in dipteran flies highlights their potential epidemiological relevance in endemic areas. Although vector competence cannot be inferred from DNA detection alone, these findings warrant further experimental research to clarify the role of *H. equina* and other forest flies in the transmission of equine piroplasmosis. Given the practicality of collecting flies—particularly in free-ranging or difficult-to-handle horses—integrating forest fly sampling into surveillance programmes may enhance EP detection in the field. Further studies combining entomological, molecular, and epidemiological approaches are needed to better define the contribution of dipteran flies to the circulation of equine pathogens.

## Figures and Tables

**Table 1:** Equine piroplasms test results for the collected forest flies (n = 170). The table includes the number of equine piroplasms-positive flies (positive/total flies collected), their species result and corresponding clade, as well as the characteristics of their hosts (sex, sampling date, and area of residence).

Horse ID	Sex	Residence Province	Sampling Date	Positive/ Total Collected Flies	Species Result	Clade
Horse 1	Mare	Segovia	21 September 2021	1/1	*Theileria equi*	E
Horse 2	Stallion	Segovia	21 September 2021	0/4	-	-
Mule 1	Molly	Segovia	21 September 2021	0/2	-	-
Horse 3	Gelding	Segovia	29 September 2021	0/2	-	-
Horse 4	Gelding	Toledo	23 August 2022	3/52	*T. equi*	A
Horse 5	Gelding	Segovia	19 September 2023	0/3	-	-
			25 September 2023	0/4	-	-
Horse 6	Gelding	Segovia	19 September 2023	1/3	*T. equi*	E
			25 September 2023	1/3	*T. equi*	E
			27 July 2024	3/3	*T. equi*	E
Horse 7	Mare	Segovia	25 September 2023	1/5	*Babesia caballi*	A
Horse 8	Mare	Segovia	25 September 2023	0/4	-	-
Horse 9	Gelding	Segovia	25 September 2023	0/5	-	-
			27 September 2023	0/18	-	-
Horse 10	Mare	Segovia	25 September 2023	0/2	-	-
			27 September 2023	0/1	-	-
Horse 11	Gelding	Madrid	23 July 2024	0/3	-	-
Horse 12	Gelding	Madrid	23 July 2024	0/2	-	-
Horse 13	Mare	Madrid	23 July 2024	0/4	-	-
Horse 14	Mare	Segovia	13 May 2024	1/2	*B. caballi*	A
			7 July 2024	1/6	*T. equi*	E
			14 July 2024	6/8	*T. equi*	E
			9 August 2024	3/8	*T. equi*	E
Horse 15	Stallion	Menorca	1 July 2024	0/1	-	-
Horse 16	Stallion	Menorca	1 July 2024	0/1	-	-
Horse 17	Mare	Menorca	1 July 2024	0/2	-	-
Horse 18	Stallion	Menorca	1 July 2024	0/1	-	-
Horse 19	Stallion	Menorca	1 July 2024	0/1	-	-
Horse 20	Stallion	Menorca	1 July 2024	0/1	-	-
Horse 21	Stallion	Menorca	2 July 2024	0/1	-	-
Horse 22	Mare	Menorca	2 July 2024	0/2	-	-
Horse 23	Mare	Menorca	2 July 2024	0/1	-	-
Horse 24	Stallion	Menorca	2 July 2024	0/2	-	-
Horse 25	Stallion	Menorca	2 July 2024	0/2	-	-
Horse 26	Mare	Menorca	2 July 2024	0/1	-	-
Horse 27	Mare	Menorca	2 July 2024	0/1	-	-
Horse 28	Mare	Menorca	2 July 2024	0/1	-	-
Horse 29	Stallion	Menorca	2 July 2024	0/1	-	-
Horse 30	Mare	Menorca	3 July 2024	0/1	-	-
Horse 31	Stallion	Menorca	3 July 2024	0/1	-	-
Horse 32	Stallion	Menorca	3 July 2024	0/2	-	-
Horse 33	Mare	Menorca	3 July 2024	0/1	-	-
Horse 34	Stallion	Menorca	3 July 2024	0/1	-	-

## Data Availability

The original contributions presented in this study are included in the article/Supplementary Materials. Further inquiries can be directed to the corresponding author.

## Supplementary Materials

The following supporting information can be downloaded at: https://www.mdpi.com/article/10.3390/pathogens15010094/s1, Table S1: Characteristics of each blood-sampled horse (n = 27), including sex, residence province, and sampling date, as well as their blood test results for EP real-time PCR, nested PCR, EP clade, and cELISA. Horses sampled in association with positive EP flies are marked with an asterisk following their identification. Figure S1. Geographical distribution of sampling sites and detection results for equine piroplasmosis (EP) by province in Spain. Pie charts represent the proportion of positive (red) and negative (green) samples for EP, as determined by nested PCR, for (A) forest flies collected from equids (n = 170) across four provinces (Madrid, Segovia, Toledo, and Menorca) and (B) horses sampled for blood analysis (n = 27) in two provinces (Segovia and Menorca). The size of the pie charts is proportional to the number of samples analysed per province.

## Abbreviations

The following abbreviations are used in this manuscript:

BLAST	Basic Local Alignment Search Tool
BOLD	Barcode of Life Data Systems
Cq	Quantification Cycle
COI	Cytochrome c oxidase subunit I
EP	Equine Piroplasmosis
MUSCLE	Multiple Sequence Comparison by Log-Expectation
NCBI	National Center for Biotechnology Information
