# Supplementary material for: Molecular Detection of Theileria equi, Babesia caballi, and Borrelia burgdorferi Sensu Lato in Hippobosca equina from Horses in Spain
Source: Pathogens. 2026 Jan 15;15(1):94. doi: 10.3390/pathogens15010094 (PMC12844906; doi:10.3390/pathogens15010094)
Supplement: Supplementary file 1 [file pathogens-15-00094-s001.zip › Table S1.pdf]

Table S1. Characteristics of each blood-sampled horse (n = 27), including sex, province, and sampling date, as well as their blood test results for EP real-time PCR, nested PCR, EP clade, and cELISA. Horses sampled in association with positive EP flies are marked with an asterisk following their identification.

Horse ID	Sex	Residence province	Sampling date	EP result real-time PCR	EP result nested PCR	EP Clade	EP result cELISA (I%)
**Horse 5**	Gelding	Segovia	25/09/2023	Negative	Negative	-	***B. caballi*** (81,41%)
**Horse 6***	Gelding	Segovia	25/09/2023	***T. equi***	***T. equi***	E	***T. equi*** (52,60%)
			05/08/2024	***T. equi***	***T. equi***	E	***T. equi*** (55,44%)<br>***B. caballi*** (56,28%)
**Horse 7***	Mare	Segovia	25/09/2023	***B. caballi***	***B. caballi***	A	***B. caballi*** (83,26%)
**Horse 8**	Mare	Segovia	25/09/2023	***B. caballi***	***B. caballi***	A	***B. caballi*** (78,35%)
**Horse 9**	Gelding	Segovia	25/09/2023	***T. equi***	***T. equi***	E	***T. equi*** (68,26%)<br>***B. caballi*** (85,04%)
			05/08/2024	Negative	Negative	-	***T. equi*** (67,83%)<br>***B. caballi*** (85,02%)
**Horse 10**	Mare	Segovia	25/09/2023	Negative	Negative	-	***B. caballi*** (55,04%)
			05/08/2024	Negative	Negative	-	Negative
**Horse 14***	Mare	Segovia	13/05/2024	***B. caballi***	***B. caballi***	A	***T. equi*** (66,75%)<br>***B. caballi*** (82,17%)
			09/08/2024	***T. equi***	***T. equi***	E	***T. equi*** (65,87%)<br>***B. caballi*** (83,85%)
**Horse 15**	OS	Menorca	01/07/2024	Negative	Negative	-	Negative
**Horse 16**	M	Menorca	01/07/2024	Negative	***T. equi***	A	***T. equi*** (78,52%)
**Horse 17**	F	Menorca	01/07/2024	Negative	***T. equi***	E	***T. equi*** (86,64%)
**Horse 18**	M	Menorca	01/07/2024	***T. equi***	***T. equi***	A	***T. equi*** (82,63%)
**Horse 19**	M	Menorca	01/07/2024	***T. equi***	***T. equi***	A	***T. equi*** (74,56%)
**Horse 20**	M	Menorca	01/07/2024	Negative	Negative	-	***T. equi*** (80,91%)
**Horse 21**	M	Menorca	02/07/2024	***T. equi***	***T. equi***	A	***T. equi*** (73,48%)
**Horse 22**	F	Menorca	02/07/2024	Negative	Negative	-	Negative
**Horse 23**	F	Menorca	02/07/2024	Negative	Negative	-	***T. equi*** (72,40%)
**Horse 24**	OS	Menorca	02/07/2024	Negative	Negative	-	Negative
**Horse 25**	OS	Menorca	02/07/2024	Negative	Negative	-	Negative
**Horse 26**	F	Menorca	02/07/2024	Negative	Negative	-	Negative
**Horse 27**	F	Menorca	02/07/2024	***T. equi***	***T. equi***	A	Negative
**Horse 28**	F	Menorca	02/07/2024	Negative	Negative	-	***T. equi* (50,01%)**
**Horse 29**	M	Menorca	02/07/2024	Negative	Negative	-	***T. equi* (73,93%)**
**Horse 30**	F	Menorca	03/07/2024	Negative	Negative	-	***T. equi* (58,21%)**
**Horse 31**	OS	Menorca	03/07/2024	Negative	Negative	-	Negative
**Horse 32**	OS	Menorca	03/07/2024	Negative	Negative	-	Negative
**Horse 33**	F	Menorca	03/07/2024	Negative	Negative	-	***T. equi*** (78,53%)
**Horse 34**	M	Menorca	03/07/2024	Negative	Negative	-	***T. equi*** (58,59%)
