# Supplementary figures and images for: Molecular Detection of Theileria equi, Babesia caballi, and Borrelia burgdorferi Sensu Lato in Hippobosca equina from Horses in Spain
Source: Pathogens. 2026 Jan 15;15(1):94. doi: 10.3390/pathogens15010094 (PMC12844906; doi:10.3390/pathogens15010094)

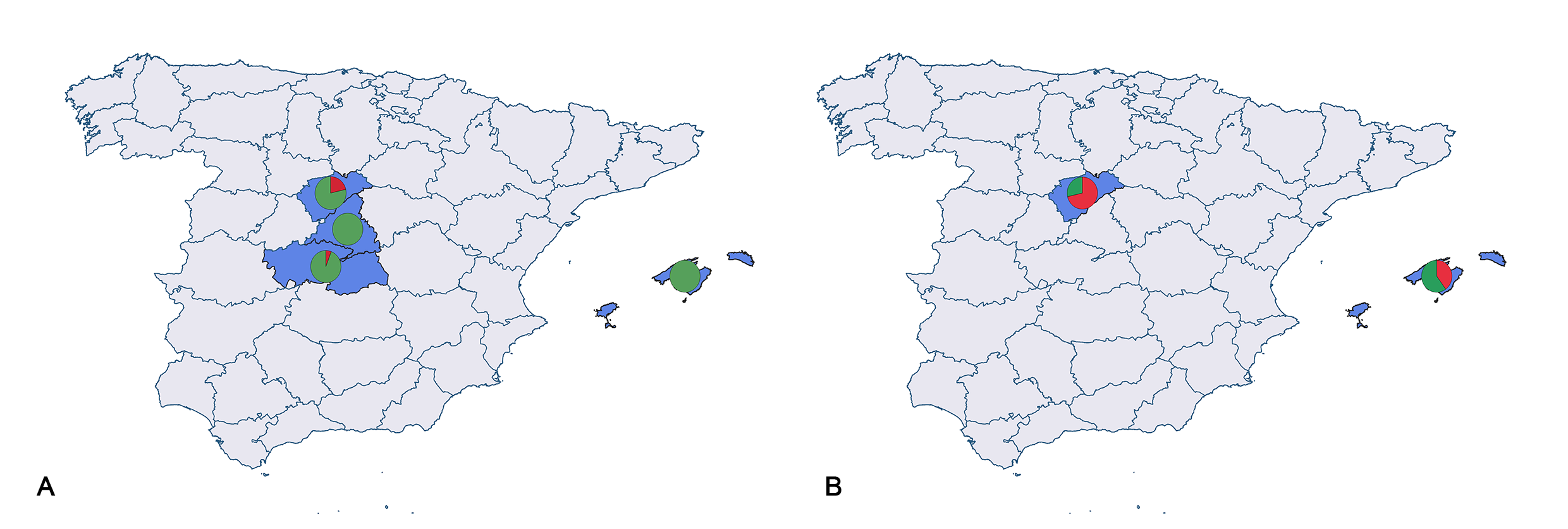

Supplement: Supplementary file 1 [file pathogens-15-00094-s001.zip › Figure S1.png]
